# Bilateral Posterior Fracture-Dislocation of the Shoulder after Electrical Shock Treated with Bilateral Hemiarthroplasty: A Case Report

**DOI:** 10.5704/MOJ.2203.025

**Published:** 2022-03

**Authors:** VA Kechagias, CA Katounis, SL Badras, I Notaras, LS Badras

**Affiliations:** Department of Orthopaedics, General Hospital of Volos, Volos, Greece

**Keywords:** bilateral shoulder posterior fracture-dislocation, electrical shock, shoulder hemiarthroplasty

## Abstract

This is a case report of a bilateral posterior fracture-dislocation of the shoulder after electrical shock and presents the first such patient treated with bilateral shoulder hemiarthroplasty. At first presentation, the upper limbs of the patient were in a position of internal rotation, and passive and active external rotations were painful. Radiographs and computed tomography of both shoulders showed bilateral posterior fracture-dislocation. Defects over 50% of the head articular surfaces led to unstable and unsuccessful initial closed and open reductions. The patient was then treated with cemented hemiarthroplasties with very good functional results two years post-operatively. This case presentation underlines the critical value of systematic clinical and radiographic evaluation of severe bilateral shoulder fracture-dislocations, prior to the ultimate proper treatment with cemented humeral shoulder hemiarthroplasties, followed by appropriate rehabilitation programme, for successful patient outcomes.

## Introduction

Bilateral posterior fracture-dislocation of the shoulder is a rare and uncommon injury. Shoulder dislocations, however, are common and account for half of all major joint dislocations and with an incidence of 10-24 cases per 100,000 people yearly^1^. Posterior dislocation is even less common accounting for 1% - 4.7% of all types of shoulder dislocations. Bilateral posterior shoulder dislocation is a rare occurrence with approximately 2.5% - 15% of all posterior dislocations. Epileptic seizure, extreme trauma and electrical shock are three major mechanisms of this entity. Electrical shock accounts for less than 5% of all bilateral posterior shoulder dislocations.

This report presents the case of a patient with rare injury of posterior fracture-dislocation of the shoulder following an electrical shock. The case signifies the importance of clinical and radiographic examinations, the appropriate treatment, and satisfactory functional post-operative patient outcomes. This is the first reported case of a patient with this rare injury treated with bilateral shoulder hemiarthroplasties.

## Case Report

**Fig. 1:**
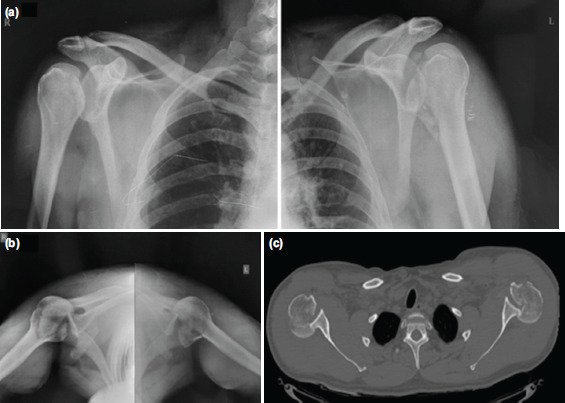
(a) Pre-operative radiographs (a) anteroposterior and (b) axillary and (c) computed tomography of both shoulders showing bilateral fracture-dislocation and defects.

A 44-year-old man, who worked as a linesman, was brought to the emergency department following an electrical shock. The patient had no past medical or surgical treatment history, no co-morbidities, no allergies, was not on any medications. Initially, he complained of pain in both hands, muscle weakness and numbness in both upper limbs, but clearly not of pain in the shoulder. So, no urgent orthopaedic referral was requested. He was monitored by the cardiology department for 24 hours during which no cardiac abnormality was noted. Then, the patient was transferred to the surgery department, where he was intravenously hydrated. It was on the second day of his hospitalisation that he complained of pain in both shoulders and was unable to move them, following which he was examined by an orthopaedist. The arms were in internal rotation. Passive and active external rotation was impossible due to pain. Shoulder radiographs in the anteroposterior (Fig. 1a) and axillary (Fig. 1b) views showed posterior fracture-dislocations of both shoulders. Computed tomography of both shoulders subsequently (Fig. 1c), confirmed bilateral posterior fracture-dislocation of the shoulders associated with a defect of over 50% head articular surface and anterior impression fracture.

**Fig. 2:**
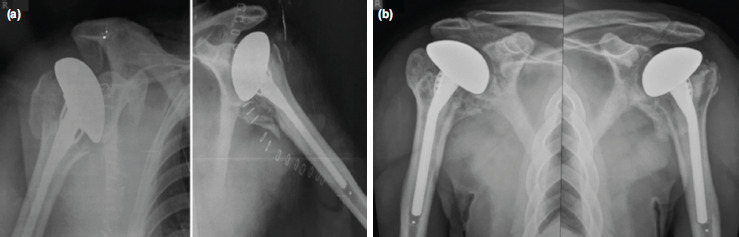
(a) Immediate post-operative and (b) two years' follow-up radiographs of both shoulders showing positions of the implants without complications.

Closed reductions were performed on the same day for both dislocated shoulders under general anaesthesia, but stable reduction of the shoulders could not be achieved. Open reduction was performed the following day, with options for stabilisation with standard implants to obtain stability or hemiarthroplasties as the final treatment. The patient was operated in a 30° beach chair position to using a deltopectoral approach. However, the open reduction was also not successful, and both reductions remained unstable. The anterior osteochondral defect of more than 50% of the articular surface was confirmed intra-operatively in both humeral heads. Thus, a decision was made to treat both shoulders with cemented hemiarthroplasties [Exacthech-Equinoxe, Florida, USA]. Cemented stems and the tuberosities were sutured to the diaphysis. Post-operative radiographs showed satisfactory positions of the implants (Fig. 2a). No complications were observed, such as paralysis of the axillary nerve, damage to the brachial plexus, injury of the axillary vessels, or rupture of the rotator cuff.

**Fig. 3:**
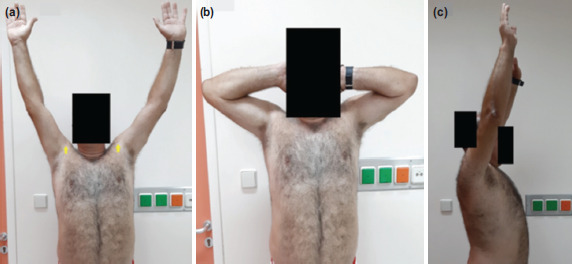
(a, b, c) Functional outcome of both shoulders two years post-operatively.

The patient used slings for both shoulders in neutral rotation and 30° - 45° abduction for four weeks. Simultaneously he underwent an intensive rehabilitation programme, according to the American Society of Shoulder and Elbow Therapists, with active and passive ranges of motion, muscle strengthening and capsular stretching exercises. The patient was examined at 3, 6 and 12 months post-operatively. At 6 months he had almost normal range of motion of shoulders and at 12 months he had complete functional recovery. No complications were observed in radiographs at two years post-operative, such as glenoid osteoarthritis, tuberosity displacement or mal-union, components malpositioning or loosening, periprosthetic fracture, joint overstuffing, rotator cuff failure, subcutaneous escape, or signs of infection (Fig. 2b). Examination of the shoulders revealed stability with normal range of motions (Fig. 3). His Constant Score was 97 on the right side and 90 on the left side. The patient experienced no pain and had returned to his daily work routines and sports.

Informed consent was obtained from the patient for data and photographs submission for publication.

## Discussion

Bilateral posterior fracture-dislocation of the shoulder, described by Munter in 1902, continues to be a rare injury. Only four cases have been reported of dislocations due to electrical shocks, and the current report is the first such patient to be treated with bilateral hemiarthroplasties^2-5^.

Management of posterior fracture-dislocation of the shoulder depends on the size of the humeral head impression defect, the type of fracture, the duration of dislocation untreated, the patient’s age and demand, occupation, and level of activity.

Thus, defects of up to 25% head articular surface can be treated by closed reduction and pin stabilisation. For defects between 25% and 50% head articular surface, or unstable joints, surgical treatment is required. Thus, subscapularis tendon transfer into the bone can be performed for unstable joints. Internal fixation is also a treatment option. For defects with over 50% head articular surface, if a three- or four-part fracture is complicated by dislocation or a case of younger patients with highly comminuted fractures, treatment with hemiarthroplasty should be considered.

Our patient was a young man, and both humeral head defects involving more than 50% of the articular surface. Closed reduction and open reduction methods were both unsuccessful with finally both shoulders having to be treated with cemented humeral shoulder hemiarthroplasties. A good rehabilitation programme achieved good results post-operatively and at two years, the patient had no pain and was satisfied with the functional result.

In conclusion, orthopaedists must be prudent when treating patients with injuries from electrical shock, as there may be fractures and dislocation of the shoulders. Doctors in the accident and emergency (A&E) department must conduct an appropriate and early clinical and radiological examination. When indicated, these injuries could be treated with shoulder hemiarthroplasties with very good results post-operatively.
